# Evaluation of undetected cases during the COVID-19 epidemic in Austria

**DOI:** 10.1186/s12879-020-05737-6

**Published:** 2021-01-13

**Authors:** C. Rippinger, M. Bicher, C. Urach, D. Brunmeir, N. Weibrecht, G. Zauner, G. Sroczynski, B. Jahn, N. Mühlberger, U. Siebert, N. Popper

**Affiliations:** 1 DWH Simulation Services, DEXHELPP, Neustiftgasse 57-59, 1070 Vienna, Austria; 2 TU Wien, Institute of Information Systems Engineering, Favoritenstraße 11, 1040 Vienna, Austria; 3 Institute of Public Health, Medical Decision Making and Health Technology Assessment, Department of Public Health, Health Services Research and Health Technology Assessment, UMIT - University for Health Sciences, Medical Informatics and Technology, Eduard-Wallnoefer-Zentrum 1, 6060 Hall i.T, Austria; 4 Division of Health Technology Assessment, ONCOTYROL - Center for Personalized Cancer Medicine, Karl-Kapferer-Straße 5, 6020 Innsbruck, Austria; 5 Institute for Technology Assessment and Department of Radiology, Massachusetts General Hospital, Harvard Medical School, 101 Merrimac St, Boston, MA 02114 USA; 6 Center for Health Decision Science, Departments of Epidemiology and Health Policy & Management, Harvard T.H. Chan School of Public Health, 718 Huntington Avenue, Boston, MA 02115 USA

**Keywords:** COVID-19, Undetected cases, Agent-based modelling

## Abstract

**Background:**

Knowing the number of undetected cases of COVID-19 is important for a better understanding of the spread of the disease. This study analyses the temporal dynamic of detected vs. undetected cases to provide guidance for the interpretation of prevalence studies performed with PCR or antibody tests to estimate the detection rate.

**Methods:**

We used an agent-based model to evaluate assumptions on the detection probability ranging from 0.1 to 0.9. For each general detection probability, we derived age-dependent detection probabilities and calibrated the model to reproduce the epidemic wave of COVID-19 in Austria from March 2020 to June 2020. We categorized infected individuals into presymptomatic, symptomatic unconfirmed, confirmed and never detected to observe the simulated dynamic of the detected and undetected cases.

**Results:**

The calculation of the age-dependent detection probability ruled values lower than 0.4 as most likely. Furthermore, the proportion of undetected cases depends strongly on the dynamic of the epidemic wave: during the initial upswing, the undetected cases account for a major part of all infected individuals, whereas their share decreases around the peak of the confirmed cases.

**Conclusions:**

The results of prevalence studies performed to determine the detection rate of COVID-19 patients should always be interpreted with regard to the current dynamic of the epidemic wave. Applying the method proposed in our analysis, the prevalence study performed in Austria in April 2020 could indicate a detection rate of 0.13, instead of the prevalent ratio of 0.29 between detected and estimated undetected cases at that time.

## Background

Undetected severe acute respiratory syndrome coronavirus-2 (SARS-CoV-2) infections play an important role in the spread of coronavirus disease 2019 (COVID-19). A simulation study estimated that, before widespread travel restrictions were put in place (January 10th – 23rd 2020), 86% of all infections in China were undetected and that up to 80% of the documented infected individuals had been infected by undetected infected individuals [1]. Infections are not recognized due to mild or absent symptoms, limited awareness of the virus in the general population, or lack of testing. The proportion of unreported cases, however, changes over time because of increased awareness, improved testing strategies and established measures changing the spread of the epidemic (isolation, self-quarantine, contact precautions, and travel restrictions). For example, in China, immediately after travel restrictions were in place (after January 23rd 2020), the fraction of all undetected infections decreased to 35% [1].

Although undetected cases of COVID-19 contribute significantly to the spread of the disease, it is still unclear how large the number of unreported cases really is. Clearly, it strongly depends on the predominant testing strategy but also on the proportion of asymptomatic cases. For the latter, estimates have been made, such as that from Iceland where 43% of the positive cases in an overall population screening reported no symptoms [2] and for passengers of the Diamond Princess resulting in an asymptomatic proportion of only 17.9% [3].

Methods commonly used to determine the number of undetected COVID-19 cases include representative studies using reverse transcription polymerase chain reaction (PCR) or antibody tests. In the first, a random sample of the general population is drawn and PCR tests are applied to identify currently infected individuals independent of current symptoms or recent exposure [4–6]. These studies provide an approximation of unreported cases, but only for a specific point in time. On the other hand, studies using antibody tests try to identify people who have been infected by SARS-CoV-2 in the past. Both types of studies are often limited due to sample size and representation of specific populations or regions and results depend on test accuracy and the time point of sample collection (when virus or antibodies are already or still detectable).

In this modeling study, we aim to determine the impact of detection probabilities on the temporal variation of the epidemic, including the fractions of undetected and detected cases of COVID-19 during an epidemic wave. We account for preventive measures in a retrospective analysis of secondary data using an agent-based model representing the entire Austrian population. We simulate different scenarios to account for the uncertainty in the proportion of undetected cases due to contradictory or lacking data. Our results can guide the interpretation of the results of prevalence studies and estimations of the proportion of undetected cases of COVID-19 in Austria.

## Methods

We used a previously published agent-based model to simulate the COVID-19 epidemic in Austria by modelling the different disease stages and events of the patient pathway [7]. Considering age-dependent detection probability, we calibrated the model to the past epidemic curve in Austria. As model results, we considered the ratio between detected and undetected cases of COVID-19 during the different phases of the epidemic.

### Agent-based simulation model

A detailed description of the model is provided by Bicher et al. [7]. Briefly, the agent-based model simulates each member of the Austrian population using statistical representatives, mapping a contact network based on different locations, such as households, workplaces, schools, and leisure time [8]. The model considers the entire disease-pathway of a COVID-19 patient with its different potential stages and events starting with the infection of healthy individuals. The model considers the time period between infection and symptom onset, as well as a “reaction-time” that covers the time between symptom onset, testing, positive test result, and the time at which the COVID-19 patient is recorded as a confirmed case in the official vigilance system. In an alternative pathway, infected individuals in the model would never be tested for COVID-19 and remain an undetected case throughout the simulation.

The time period from February 19th 2020 to June 10th 2020 is simulated and reported outcomes include the number of detected and undetected cases in each of the different disease stages for each point in time. In the model, four groups of infected individuals are distinguished: *presymptomatic*, *symptomatic unconfirmed*, *confirmed* and *never detected*. *Never detected* individuals will never be tested for COVID-19. Their disease course is modeled over 9.4 days in total, accounting for the time from infection until the infectious period and the time of the infectious period. P*resymptomatic*, *symptomatic unconfirmed*, *confirmed* individuals receive a positive test result at some point in time during the simulation. *Presymptomatic* individuals are still in the incubation period and *symptomatic unconfirmed* individuals have started to experience symptoms but have not yet received a positive test result. Finally, the *confirmed* cases are recorded until their recovery, considering different time periods depending on the severity of the disease. Undetected cases at a given time consist of *presymptomatic*, *symptomatic unconfirmed* and *never detected*. Fatal COVID-19 infections are not investigated for this analysis and are counted as recovered cases. Confirmed cases are classified into several disease severities: mild cases which can recover at home, severe cases which require hospitalization, and critical cases which require treatment at an intensive care unit (ICU). Table 1 provides an overview of the parameter values for the mentioned time periods.

**Table 1:** Parameter values for the model

	Value	Reference
Incubation period	5.1 days (1.78)	Lauer et al. [9]
Latency period	2 days	Robert Koch Institute [10]
Time until infectious period	3.1 days (1.78)	Incubation period minus latency period
Infectious period	6.3 days (1.25)	Expert estimates in line with Robert Koch Institute [10]
Delay from symptom start to positive test result	3.8 days (2.38)	Hellewell et al. [11]
Time between positive test result and recovery for detected mild cases	13.3 days (2.46)	Based on reported COVID-19 data in Austria (Epidemiologisches Meldesystem [12])
Time between positive test result and recovery for detected severe cases	20.0 days (11.75)	Based on reported COVID-19 data in Austria (Epidemiologisches Meldesystem [12])
Time between positive test result and recovery for detected critical cases	25.0 days (9.09)	Based on reported COVID-19 data in Austria (Epidemiologisches Meldesystem [12])

Data are mean (standard deviation). The latency period is assumed to be a fixed value

### Age-dependent detection probability

The detection probability *θ* determines whether an infected individual will be tested positive for COVID-19. It combines the probability of developing symptoms specific enough to get tested for COVID-19, and the probability of being detected because of other testing strategies, such as screening of care homes. We made the simplifying assumption that *θ* remains constant for all age-groups during our considered time interval (March to June 2020) but is in fact age dependent due to reported evidence of increasing probability to develop more severe symptoms with increasing age [13].

To determine the age-dependent detection probability, we first calculated the cumulative incidence of confirmed cases in Austria $\upsigma =\frac{\# confirmedCases}{\# population}$ as of May 6th 2020. We then assumed that all age groups have been affected equally by the disease and that the mismatch between the age distribution of the confirmed cases and the Austrian age pyramid is caused by an age-dependent detection rate *θ*_*i*_. Considering 10-year age groups, we calculate

1 $$ \overset{\sim }{\uptheta_i}=\frac{c_i\cdotp \uptheta}{p_i\cdotp \upsigma}, $$

whereby *c*_*i*_ denotes the cumulative number of confirmed cases of this age group and *p*_*i*_ denotes the total population of this age group. For high values of *θ*, some age groups, especially the elderly people, are overrepresented, resulting in a value *θ*_*i*_ > 1. Therefore, we finally compute

2 $$ {\uptheta}_i=\mathit{\min}\left(\overset{\sim }{\uptheta_i},1\right). $$

## Calibration

Model calibration is the process of adjusting model parameters to match the data observed in the real world, in this case confirmed COVID-19 cases. To achieve this goal, we adjust the detection probability and the infection probability in case of contact.

In our model, increasing the detection probability parameter has two major consequences: (a) the number of confirmed cases in the model at a given point in time increases because more cases are detected, and (b) the number of confirmed cases is decreasing over time because there are fewer undetected cases causing uncontrolled infections throughout their whole infectious period without being quarantined. Decreasing the detection probability has the opposite effect. Therefore, the infection probability is adjusted whenever the detection probability is changed to keep the number of confirmed cases at the desired level.

To account for the lack of information considering *θ*, the calibration of the infection probability has been performed for different values of *θ* ranging from 0.1 to 0.9 to reproduce the epidemic curve of COVID-19 cases in Austria [14]. This calibration process has been split into two parts: calibrating the infection probability α_1_ and *α*_2_ before and after the peak of the epidemic wave. In the first part, the infection probability α_1_ is calibrated using a bisection algorithm with a Monte-Carlo Simulation in the loop to reproduce the officially reported number of confirmed cases at the peak of the epidemic wave. In the second part, *α*_2_ is adjusted using the same bisection algorithm to reproduce the decrease of the officially reported number of positive COVID-19 cases in Austria until June 10th 2020. The lockdown measures are modelled equivalently and as described by Bicher et al.^7^ and include school closure, increased home office use, leisure time contact reduction, and increased hygienic measures starting on March 16th 2020 and being gradually lifted from April 16th 2020 onwards.

### Actual detection rate and interpolation

While the detection probability *θ* is a model input, the observed detection rate *ϑ* can be obtained as a model output by evaluating

3 $$ \vartheta =\frac{\# confirmedCases}{\# allInfectedCases} $$

at the end of the simulation. To clearly differentiate between the input parameter *θ* and the observed model output *ϑ*, we henceforth denote the first as detection probability and the second as detection rate. The detection rate relies heavily on the detection probability. Therefore, the model results for different assumptions of *θ* can be expressed as a function of *ϑ*. Assuming the model output reacts steadily to changes in *θ*, model output for input parameters *θ* in between the directly calibrated input parameters can be interpolated using piecewise linear splines.

The model was developed, calibrated, and analyzed following the International Society for Pharmacoeconomics and Outcomes Research – Society for Medical Decision Making (ISPOR-SMDM) Modeling Good Research Practices guidance [15].

## Results

Age-dependent detection probabilities *θ*_*i*_ given an overall detection probability *θ* calculated via (1) are provided in Table 2. As expected, with increasing *θ*, all age-specific *θ*_*i*_ increase as well with younger age groups having lower values than older age groups. It can be seen that *θ* lower than 0.4 leads to no overrepresented age groups, that is age classes *i* with $\overset{\sim }{\uptheta_i}>1$ (compare (2)).

**Table 2:** Values for ***θ***_***i***_ for a given ***θ***

	*θ*_0_	*θ*_10_	*θ*_20_	*θ*_30_	*θ*_40_	*θ*_50_	*θ*_60_	*θ*_70_	*θ*_80_	*θ*_90_
*θ* = 0.1	0.02	0.04	0.11	0.10	0.12	0.14	0.10	0.10	0.16	0.29
*θ* = 0.2	0.03	0.09	0.22	0.20	0.24	0.28	0.20	0.21	0.33	0.58
*θ* = 0.3	0.05	0.13	0.32	0.29	0.36	0.41	0.30	0.31	0.49	0.87
*θ* = 0.4	0.06	0.17	0.43	0.39	0.48	0.55	0.40	0.41	0.65	1.00^(1)^
*θ* = 0.5	0.08	0.21	0.54	0.49	0.59	0.69	0.50	0.51	0.82	1.00^(1)^
*θ* = 0.6	0.09	0.26	0.65	0.59	0.70	0.83	0.59	0.62	0.98	1.00^(1)^
*θ* = 0.7	0.11	0.30	0.75	0.69	0.83	0.97	0.69	0.72	1.00^(1)^	1.00^(1)^
*θ* = 0.8	0.12	0.34	0.86	0.79	0.95	1.00^(1)^	0.79	0.82	1.00^(1)^	1.00^(1)^
*θ* = 0.9	0.14	0.39	0.97	0.88	1.00^(1)^	1.00^(1)^	0.89	0.93	1.00^(1)^	1.00^(1)^

***θ***_***i***_ denotes the age-dependent detection probability for people within the age group [i, i+ 9]. ***θ***_**90**_ denotes the age-dependent detection probability for people aged 90 or older. Cells marked with ^**(1)**^ indicate that the age class is overrepresented with $\overset{\sim }{{\boldsymbol{\uptheta}}_{\boldsymbol{i}}}>\mathbf{1}$ and were truncated to one via (2)

Calibrated infection probabilities gained by fitting the simulation results to the epidemic wave in Austria from March 11th 2020 to June 18th 2020 for different assumptions of the overall detection probability *θ* are shown in Table 3. Results indicate that the impact of a varied detection probability on the confirmed cases can be adjusted with small variation of the infection probability *α*_1_ and *α*_2_ before and after the observed peak of the confirmed cases.

**Table 3:** Calibrated values for the infection probability ***α***_**1**_ and ***α***_**2**_ for different detection probabilities ***θ***

	*θ* = 0.1	*θ* = 0.2	*θ* = 0.3	*θ* = 0.4	*θ* = 0.5	*θ* = 0.6	*θ* = 0.7	*θ* = 0.8	*θ* = 0.9
Infection probability before peak *α*_1_	5.960%	5.862%	5.840%	5.840%	5.850%	5.960%	6.030%	6.032%	6.047%
Infection probability after peak *α*_2_	5.215%	4.502%	4.526%	4.830%	5.148%	5.394%	5.729%	6.394%	6.555%
Observed detection rate *ϑ*	0.09	0.18	0.27	0.37	0.45	0.55	0.63	0.70	0.75

Each parameter value triple (**θ**, **α**_**1**_, **α**_**2**_) is specified to lead to the same curve for the confirmed infected individuals. The row ***ϑ*** displays the actual detection rate evaluated by (3) at the end of the simulation

**Fig. 1:**
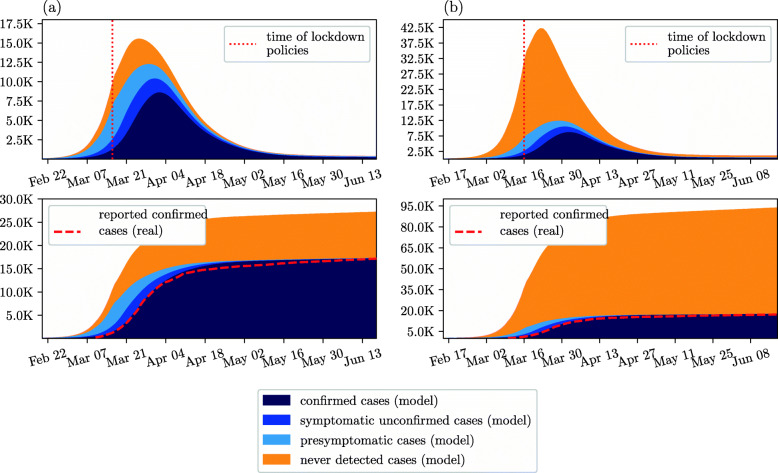
Visualization of the first epidemic wave for detection rate ***ϑ*** =0.63(a) and ***ϑ*** =0.18(b)

**Fig. 2:**
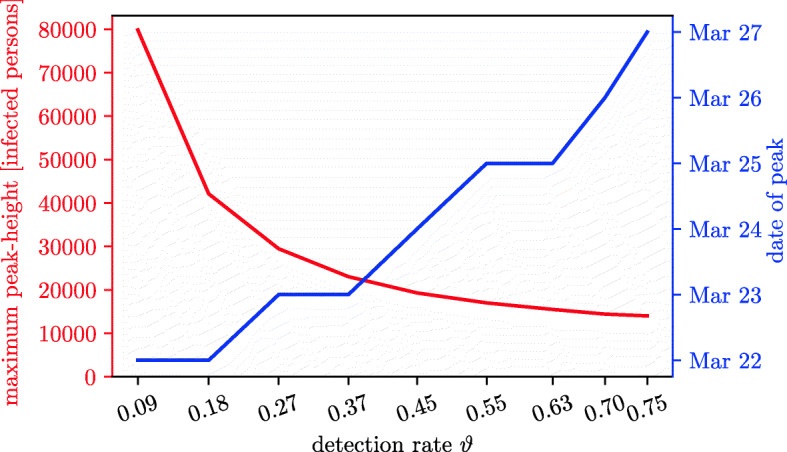
Height and date of the real peak as a function of the detection rate ***ϑ***

**Fig. 3:**
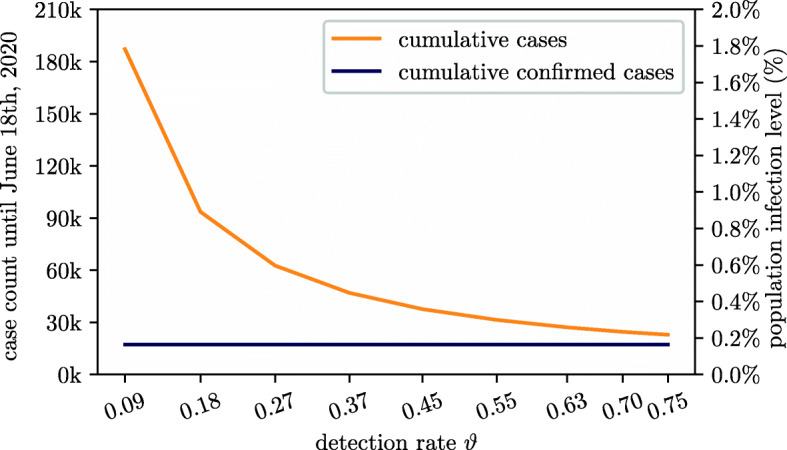
Cumulative number of total simulated (detected and undetected) and confirmed (17,175) infected individuals until 2020 June 18th as a function of the detection rate ***ϑ***. The right-hand-side axis displays the values in percent of the total Austrian population (8,901,064) on 2020.01.01 according to Statistics Austria

Figure 1 shows the simulated results for the epidemic wave in Austria from March 11th 2020 to June 18th 2020 for two exemplary values of *ϑ*, distinguishing the four different groups of infected individuals mentioned in section “Methods”. Each group of infected individuals peaks at a different point in time due to different disease durations and sequences within the disease pathway. In particular, the peak of the sum of all groups, which we will denote as the *real peak*, occurred a few days before the peak of the confirmed cases, the *official peak*, on April 2nd. Figure 2 visualizes how the real peak would change with increasing the detection probabilities. The left axis displays the number of cases at the real peak, the right axis its date. It shows that increasing the detection rate leads to a lower and earlier real peak. Figure 3 displays the cumulative number of infected individuals until June 18th 2020 depending on the detection rate, which directly correlates with the cumulative incidence in the population. With unchanged number of cumulative confirmed cases (lower curve) the overall cumulative cases (upper curve) decreases with the detection probability, leading to a lower cumulative incidence.

**Fig. 4:**
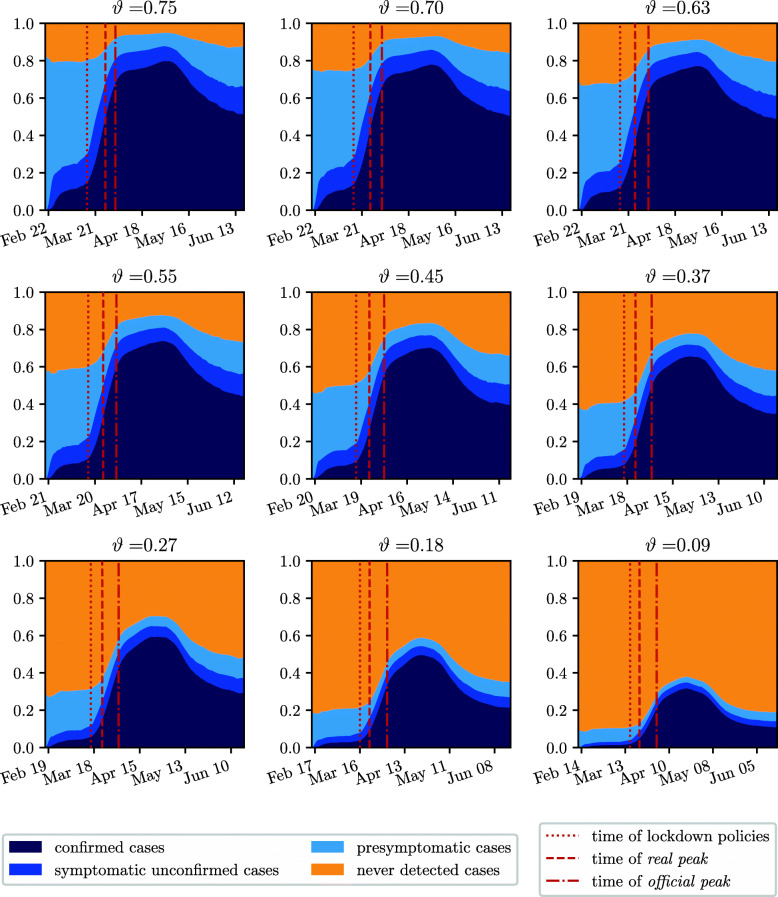
Time dependent split of the total infected population into the separate disease states displayed for different detection rates ***ϑ***

**Fig. 5:**
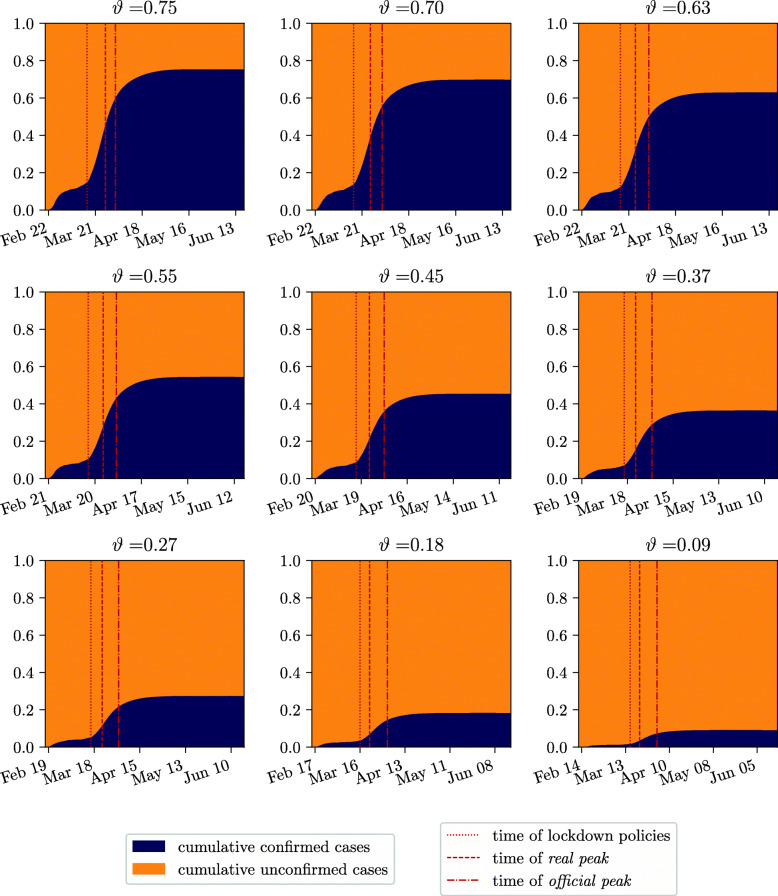
Time dependent split of the cumulative infected population into detected and undetected cases for different detection rates ***ϑ***

Figure 4 shows how the ratio between currently infected detected and undetected cases changes during the epidemic. In the upswing phase, the detected cases account for a very small part of all the infected cases, whereas their share increases rapidly with the start of the lockdown policies on March 16th. The share of confirmed cases increases more slowly at the time of the *official peak* and decreases again in the “steady-state phase” at the end of the epidemic wave. Figure 5 shows the same for the cumulative detected and undetected cases. Again, the cumulative detected cases account for only a small proportion of all cumulative infected cases during the upswing phase of the epidemic, before this proportion reaches a constant state a few days after the *official peak.*

**Fig. 6:**
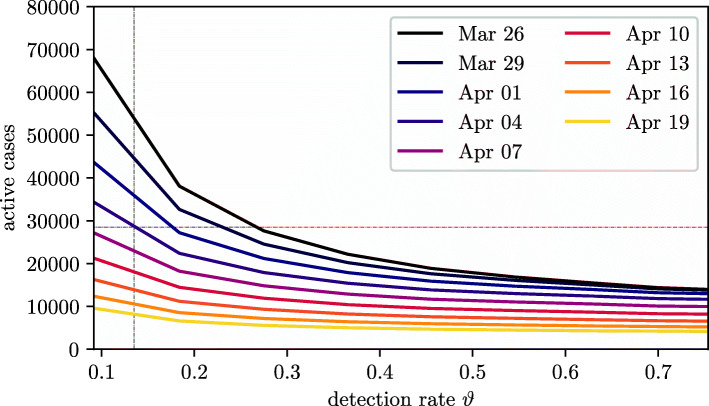
Number of active cases for different dates as a function of the detection rate. The dashed line represents the 28,500 cases on April 4th as estimated by SORA Institute for Social Research and Consulting, leading to ***ϑ*** = 0·13

Figure 6 illustrates how the model results can be used to interpret the findings of studies identifying currently infected individuals. It displays the number of active cases of COVID-19 for different dates as a function of the detection rate. Given an estimate of active cases for a specific date, the model results for this date as a function of the detection rate can be interpolated by linear splines and the detection rate matching the estimate of active cases can be determined.

## Discussion

The ratio between detected and undetected cases strongly depends on the phase of the epidemic. Results of randomized screenings to evaluate the detection rate therefore need to be interpreted within the context of the phase of the epidemic. This holds for both studies using antibody tests and PCR tests.

Screening programs using antibody tests are less sensitive but still depend on timing within the epidemic. The different severities between detected and undetected cases lead to different disease lengths. Therefore, the confirmed cases are underrepresented within the total cases as long as the disease wave is still on the upswing (see Fig. 5). Consequently, results of antibody screenings would underestimate the detection rate, if performed too early before the completion of the wave. Similarly, considering the indication of waning antibody, screenings performed too long after an epidemic wave could underestimate the total number of COVID-19 infections, and therefore, overestimate the detection rate.

As for studies using PCR tests to determine the currently infected individuals, the simulation results show that the actual detection rate cannot be determined without additional information about the disease wave. An observed ratio of 60:40 for detected vs. undetected cases in a randomized trial might indicate *ϑ* =0.37 if the study was performed a couple of days after the official peak, or *ϑ* =0.75 if it was performed a couple of days before. Therefore, the disease progression should always be considered retrospectively when evaluating and interpreting a study performed to determine the detection rate.

In Austria, three different nationwide randomized prevalence screening programs have been performed to get an image of current prevalence [6]. The first program was organized by SORA Institute for Social Research and Consulting with tests between April 1st and 6th (sample size *n* = 1544, 6 positive tests) [4], the other two programs were executed by the Austrian Statistics Institute between April 22nd to April 25th (sample size *n* = 1432, 1 positive test result) and May 26th to May 30th (sample size *n* = 1279, no positive test results). As indicated by Fig. 4, the first and second study were executed at a time with the lowest possible fraction of undetected cases possible: shortly after the *official peak* of the epidemic wave. Consequently, a high sample size would be required to detect a significant number of undetected cases and even then the resulting ratio needs to be treated with care: For example, an actual detection rate of *ϑ* =0.18 results in a prevalence of almost 50% detected cases on April 10th. With respect to the ratio between undetected and detected, the timing of the third study was much better suited. Unfortunately, the total number of infected individuals at the end of May was extremely low which, again, required a large sample size. As a result of the unfortunate timing and the limited number of participants, the results of all three studies in Austria have not been particularly useful in providing conclusions about the detection rate parameter. Yet, the combination of the connected surveys brought very interesting insights into the actual wellbeing of the population.

Nevertheless, taking the dynamic of the epidemic wave into consideration, the first PCR study in Austria performed by SORA in April (sample size *n* = 1544) [4] could be used to determine feasible values for the detection probability. The study estimates a prevalence of 28,500 infected persons in Austria for April 4th. As demonstrated in Fig. 6, this leads to *ϑ* =0.13. Note that this number is considerably lower than the prevalent ratio between detected and undetected 8358/28,500 ≈ 0.29 on this day [14]. Consequently, a large error is made when estimating the detection rate based on this naïve formula.

The graphs in Fig. 4 in combination with Fig. 1 indicate that there are points in time much better suited for randomized prevalence studies, namely the time of the *real peak* which occurred about 5 to 10 days after the lockdown measures and 5 to 10 days before the *official peak*. At that time, the overall number of infected individuals is high, thus the required sample size is reduced. But the detection rate is still comparably low which increases the chances of including undetected individuals in the screening sample. Unfortunately, this is a rather theoretical result, as the time of the *real peak* can only be evaluated retrospectively. Nevertheless, it indicates that the optimal time for prevalence studies lies during the upswing of the epidemic wave, and that they should not be performed too late.

Moreover, the data preprocessing required for parametrization provides new insights into the level of detection probabilities. As displayed in Table 2, probabilities higher than 0.4 cannot be age-stratified using the proposed algorithm indicating this value as an upper bound for the detection probability in Austria between March and June 2020. These insights concur with several publications [5, 16, 17] who all suggest a comparably low detection probability far smaller than 50%, but contradicts the results of several screening programs performed earlier in China [3] and Iceland [2] which all resulted in higher detection rates. Yet, it is not surprising that detection probabilities differ between different countries. Although evaluated for Austria, the qualitative results of our study can be applied to any country or region worldwide if we assume that the testing strategy regarding the target groups, the sensitivity and specificity of the tests, and the testing and reporting delays are similar.

The calculation of the age-dependent detection probability also has an influence on the detection rate ϑ as a model output. One would expect that the detection rate ϑ should be identical to the detection probability θ. Instead, ϑ < θ can be observed, with the difference increasing with larger values for θ (see Table 3). This effect occurs because the simulation model is not able to reproduce the large number of infected elderly people observed in reality. For large values of θ, this is mainly caused by capping the age-dependent detection probability at 1. But it is also a result of the implemented contact network for retired people. They often live in smaller households and do not have any workplace contacts, making them less likely to be in contact with an infected individual.

Still, the contact network is one of the key features of our simulation model. It is based on the POLYMOD study [8], a large survey on social contact behavior relevant to the spread of infectious diseases. In addition, the location-based contact network allows us to implement specific lockdown policies such as school closures directly without the need of “guessing” parameter values for their implications. Even if this model feature is not directly needed for evaluating the number of undetected cases of COVID-19, it allows the simulation of the epidemic wave as realistically as possible. A feature much more important for the analysis presented in this study is that the agent-based model includes undetected cases of COVID-19 in the spread of the disease, and a snapshot can be taken of all the infected individuals for every timestep, separating them into their respective disease states.

Our study has several major limitations. First, the calculation of the age-dependent detection probability relies on the simplifying assumption that people of all age groups are equally susceptible to getting infected and that the mismatch between the age distribution of the confirmed cases and the overall Austrian age distribution is solely due to an age-dependent probability to develop symptoms and subsequently getting tested for the disease. This subject is strongly linked to the debate on whether schools play a major role in the spread of COVID-19. Since the scientific community could not yet reach a consensus on the latter [18, 19], we did not assume that children are less susceptible to becoming infected.

Second, we kept the age-dependent detection probability constant over time. Thus, we did not account for screening programs performed in care homes or the time during which schools were closed and children could only be infected by their immediate family. Moreover, we did not model the sensitivity and the specificity of the current PCR test for SARS-CoV-2 or the increased usage of antigen tests whose performances vary greatly regarding their sensitivity and specificity. Our model does not provide the possibility for an uninfected individual to receive a false positive test result, and an infected individual who gets tested for the disease will always receive a positive test result in our analysis. This way, in our model, the sensitivity of the test is implicitly incorporated into the detection probability θ with a lower test sensitivity producing a lower detection probability.

Furthermore, as for all current simulation models for COVID-19, the parameters used in our model are associated with uncertainty. In particular, the recovery time for undetected cases – that is, the time until these mostly asymptomatic and mildly symptomatic individuals are no longer infectious – is highly uncertain. This is certainly an influential parameter in the model but real data and evidence for its values are diverse and hard to find. We also have not modelled a varying infectiousness depending on the severity of the disease or the time elapsed since the start of the infectious period. Instead, all agents are assumed to be equally infectious throughout the infectious period. Current studies suggest that this is likely not the case, and therefore our assumption constitutes a major simplification. Although our simulation model allows us to include infectiousness varying across individuals, we decided against the implementation of infectiousness heterogeneity, as respective data are lacking. On the same note, our model does not specifically consider super-spreader events caused by an overly infectious person or an event where one infected person has contact with an extraordinarily high number of people. However, the detection probability is independent of the origin of the infection (caused by a super-spreader event or not) and the ratio of detected and undetected cases of COVID-19 remains unaffected. Therefore, not specifically considering super-spreader events from the simulation does not bias the results of our study.

Lastly, our simulation model does not give an estimate for the fatal cases. The case fatality ratio (CFR) of COVID-19 strongly depends on the need and the availability of medical resources, namely the ICU-beds and medical personnel. Since this number of total ICU-beds and human resources available for COVID-19 patients in Austria is not fixed but can be slightly adapted to the current need, our model does not consider a threshold for the available medical resources and a subsequential change in the CFR as soon as the maximal capacities are exceeded. Therefore, an estimation of the fatal cases is associated with great uncertainty, and even more importantly, provides no added value to the model results of the current research question of our study.

In conclusion, we presented the outcomes of a retrospective modeling study in which we compared different assumptions about the probability of detecting an individual infected by SARS-CoV-2, leading to different “dark figures”, which allowed us to establish possible value ranges for this relevant disease parameter.

Moreover, our simulation outcomes display that the ratio between confirmed and unconfirmed cases for the Austrian COVID-19 epidemic heavily changes with time. Consequently, the results of randomized screenings are usually interpreted too naively. This study gives guidance on when to setup and how to interpret screening results in a more accurate way.

## Data Availability

The datasets used and/or analysed during the current study are available from the corresponding author on reasonable request.

## Abbreviations

SARS-CoV-2: Severe acute respiratory syndrome coronavirus-2
COVID-19: Coronavirus disease 2019
PCR: Reverse transcription polymerase chain reaction test
ICU: Intensive care unit
ISPOR-SMDM: International Society for Pharmacoeconomics and Outcomes Research – Society for Medical Decision Making
SORA: Institute for Social Research and Consulting
CFR: Case fatality ratio
